# Delta Neutrophil Index as a Marker for Differential Diagnosis between Acute Graft Pyelonephritis and Acute Graft Rejection

**DOI:** 10.1371/journal.pone.0135819

**Published:** 2015-08-14

**Authors:** Dong Ho Shin, Eun Jung Kim, Soo Jin Kim, Ji-Young Park, Jieun Oh

**Affiliations:** 1 Department of Internal Medicine, Kangdong Sacred Heart Hospital, Hallym University, Seoul, Korea; 2 Department of Laboratory Medicine, Kangdong Sacred Heart Hospital, Hallym University, Seoul, Korea; 3 Department of Internal Medicine, Kangdong Sacred Heart Hospital, Hallym Kidney Research Institute, Hallym University, Seoul, Korea; University of Toledo, UNITED STATES

## Abstract

**Introduction:**

The delta neutrophil index (DNI) is the fraction of circulating immature granulocytes, which reflect infectious and/or septic condition. Acute graft pyelonephritis (AGPN) versus acute graft rejection is a frequently encountered diagnostic and therapeutic dilemma in kidney transplant recipients, but little is known about the clinical usefulness of DNI value in the differentiation of the two conditions.

**Material & Methods:**

A total of 90 episodes of AGPN or acute graft rejection were evaluated at the Kangdong Sacred Heart Hospital between 2008 and 2014. We performed retrospective analysis of demographic, clinical, and laboratory parameters data. Receiver operating curves (ROC) and multivariate logistic regression were conducted to ascertain the utility of DNI in discriminating between AGPN and acute graft rejection.

**Results:**

AGPN group had significantly higher DNI values than acute graft rejection group (2.9% vs. 1.9%, P < 0.001). The area under the ROC curve for DNI value to discriminate between AGPN and acute graft rejection was 0.85 (95% confidence interval [CI]; 0.76–0.92, P < 0.001). A DNI value of 2.7% was selected as the cut-off value for AGPN, and kidney transplant recipients with a DNI value ≥ 2.7% were found to be at a higher risk of infection than those with a DNI < 2.7% (odd ratio [OR] 40.50; 95% CI 8.68–189.08; P < 0.001). In a multivariate logistic regression analysis, DNI was a significant independent factor for predicting AGPN after adjusting age, sex, log WBC count, log neutorphil count, log lymphocyte count, CRP concentration, and procalcitonin concentration (OR 4.32; 95% CI 1.81–10.34, P < 0.001).

**Conclusions:**

This study showed that DNI was an effective marker to differentiate between AGPN and acute graft rejection. Thus, these finding suggest that DNI may be a useful marker in the management of these patients.

## Introduction

Acute graft pyelonephritis (AGPN) is a common form of bacterial infection in kidney transplant recipients and acute graft rejection frequently encounter in those people [1–3]. However, sometimes the distinction between AGPN and acute graft rejection in the clinical setting is difficult [1]. AGPN is defined as the simultaneous presence of fever and a urine culture showing bacterial growth, along with one or more of the following: graft pain, chills, and/or cystitis [4]. However, fever and graft tenderness can occur in acute rejection. On the other hand, immunosuppression therapy to prevent rejection may blunt these signs in AGPN [1]. Additionally, microbiologic cultures take at least 24–72 hours for adequate growth [5]. Although several biomarkers are used to aid rapid identification of bacterial infection such as C-reactive protein (CRP), procalcitonin, and various interleukins, these markers have limitations in a lack of specificity or cost-effectiveness [6–9]. Although graft kidney biopsy is the gold standard method to discriminate between AGPN and acute graft rejection, it is not always recommended or may even be contraindicated in cases of acute kidney infection.

The presence of immature granulocytes (IGs), including metamyelocytes, myelocytes and promyelocytes, as well as the granulocytic shift to the left in peripheral blood, usually reflect the enhanced production of granulocytes in bone marrow, as a result of bacterial infection [10,11]. Although several studies have also demonstrated that the percentage of IGs count can be used as an indicator of bacteremia or sepsis [12,13], determining the IGs count is laborious, and its reproducibility is highly dependent on the examiner’s technique [10,14].

The delta neutrophil index (DNI) is a novel value designed to determine the fraction of IGs more readily and reliably. It is calculated by an automatic hematologic analyzer ADVIA2120 (Siemens Healthcare Diagnostics) using myeloperoxidase (MPO) and nuclear lobularity channels in the test system. The difference between the leukocyte differentials measured in the two channels is designated as the DNI, which correlates with the IGs fraction in peripheral blood calculated by manual counting [15]. Results from some studies have shown that, compared with white blood cell (WBC) count or CRP concentration, the DNI value is a more useful marker for predicting mortality in patients with sepsis [16]. Moreover, a high DNI value appears to be an independent predicative marker for infection in febrile SLE patients, suggesting that DNI may be a useful marker for differential diagnosis between SLE flares and infection in SLE patients presenting with fever [17]. However, little is known about the clinical usefulness of DNI in the differential diagnosis of AGPN and acute graft rejection among kidney transplant recipients. Therefore, in this study, we aimed to investigate the utility of DNI in discriminating between AGPN and acute graft rejection.

## Materials and Methods

### Patients

We conducted a retrospective cohort study at Kangdong Sacred Heart Hospital, which is a 600-bed teaching hospital where 472 kidney transplants have been performed in the last 30 years. Between January 2008 and February 2014, kidney transplant recipients who had acute graft rejection or AGPN, those whose DNI values were checked, and those who were older than 18 years were considered eligible in this study. There were 45 kidney transplant recipients who had 51 acute graft rejection episodes and 49 kidney transplant recipients who had 54 AGPN episodes, respectively. Of these episodes, 15 episodes were excluded because those episodes occurred in recipients who had neutropenia resulting from drug-induced bone marrow suppression (n = 4), those episodes occurred in recipients who had other infections such as pneumonia and enteritis (n = 5), and those episodes occurred in recipients whose procalcitonin levels were not checked (n = 6). After these exclusions, 75 kidney transplant recipients with 90 episodes were enrolled in this study. At the time the episodes occurred, there were no occurrence of other diseases such as polyoma virus infection, CMV infection, and recurrent native kidney disease.

### Ethics statement

This study was carried out in accordance with the Declaration of Helsinki and approved by the Institutional Review Board (IRB) of the Kangdong Sacred Heart Hospital (Ref. 14-2-46). None of the transplant donors belonged to a vulnerable population or were subject to coercion. All patients participating in the current study were aware of this investigation. However, because this was a retrospective medical record-based study and the subjects were de-identified, the IRB waived the need for written consent from the patients.

### Definition

AGPN was defined as the simultaneous presence of fever and a urine culture showing bacterial growth and/or bacteremia along with one or more of the following: graft pain, chills, and/or cystitis [4]. Urine specimens generally are obtained by a midstream clean-catch technique. Bacterial growth were defined as bacterial growth >10^4^ colony-forming units (CFU)/mL. Symptoms of cystitis were as follows: a strong and persistent urge to urinate, a burning sensation when urinating, passing frequent and small amounts of urine, discomfort in the pelvic area, and a feeling of pressure in lower abdomen. Kidney allograft dysfunction was defined as increase in serum creatinine concentration of 25% over baseline levels. The kidney allograft outcomes were evaluated at one month after starting antibiotic therapy in AGPN patients with kidney allograft dysfunction. The outcome was defined as follows: good, > 25% reduction in serum creatinine; moderate, ≤ 25% reduction in serum creatinine; poor, no reduction in serum creatinine. Acute graft rejection was suspected in recipients with established kidney allograft function who experienced within 1–2 days a rapid increase in their plasma creatinine concentration of 10–25% over baseline levels with or without decreased urine output, graft tenderness, or fever in the absence of other obvious causes of acute renal allograft dysfunction [18]. Acute graft rejection was diagnosed by histological examination.

### Blood sampling and DNI measurement

Blood samples for the analyses of DNI were drawn from each patient into EDTA tube, and were immediately transported at room temperature to the chemical laboratory department, and the assay was performed within one hour of blood sampling.

DNI is included as part of the routine complete blood count tests at our institution. DNI calculation was carried using an automatic cell analyzer (ADVIA 2120 Hematology System, Siemens Healthcare Diagnostics, Forchheim, Germany) [15]. After red blood cell lysis, cell size and stain intensity were measured by the tungsten-halogen-based optical system of the MPO channel to count and differentiate granulocytes, lymphocytes, and monocytes based on their size and MPO content. This was followed by cell counting and classification according to size, lobularity, and nuclear density, using the laser diode-based optical system of the lobularity nuclear density channel counted. DNI value was calculated using the following formula: DNI = [the neutrophil and the eosinophil subfractions measured in the MPO channel by a cytochemical MPO reaction]—[the polymorphonuclear neutrophil (PMN) subfraction measured in the nuclear lobularity channel by the reflected light beam]. The unit of DNI value was %.

### Data collection

Demographic and clinical data such as age, gender, cadaveric or living related donor, the duration of the episodes from the time of kidney transplant, and symptoms from patients presenting with AGPN or acute rejection were recorded at the time of admission. In addition, laboratory parameters such as serum hemoglobin concentration, WBC count, neutrophil count, lymphocyte count, DNI value, platelet count, CRP concentration, procalcitonin concentration and creatinine concentration were also measured at the time of admission.

### Immunosuppressive regimens

All patients were initially maintained on a triple regimen including cyclosporine (10 mg/kg/day, adjusted to a target level of 100–150 ng/mL) or tacrolimus (0.1 mg/kg/day, adjusted to a target level of 10–15 ng/mL for the first month and 5–8 ng/mL for maintenance); prednisone (1 mg/kg/day and tapered 5 mg/week); and azathioprine (1.5 mg/kg/day) or mycophenolate mofetil (MMF) (0.5–2 g/day).

### Statistical analysis

Statistical analyses were performed using SPSS 19.0 (SPSS Inc., Chicago, Illinois, USA) and MedCalc 14.12.0 software (MedCalc Software, Acacialaan, Ostend, Belgium). Continuous variables were expressed as mean ± SD and categorical variables as numbers (percentages). Differences between two groups were assessed using the Student’s *t* test, χ², or Fisher’s exact test. The Kolmogorov-Smirnov test was used to analyze the normality of the parameter distribution. Nonparametric variables were expressed as median and range and compared using the Mann-Whitney test. The predictive value of various parameters for AGPN was analyzed by receiver operating characteristic (ROC) curve analysis and the area under the ROC curve (AUC) was calculated. Analysis of independent predictive parameters for AGPN was ascertained by multivariate logistic regression analysis, which included all covariates with a P-value < 0.1 on univariate analysis.

## Results

### AGPN and acute graft rejection episodes

Forty-six kidney transplant recipients presented with 52 episodes of AGPN during the study period as follows one episode: 40 patients; two episodes: six patients. Out of the 52 episodes, there were seven episodes with bacteremia. In addition, 29 kidney transplant recipients presented 38 episodes of acute graft rejection during the study period as follows one episode: 20 patients; two episodes: nine patients. Among acute graft rejection episodes, 19 episodes of T-cell mediated rejection, four episodes of antibody mediated rejection, and 15 episodes of borderline change were proven by biopsy.

### Clinical characteristics and comparison of patients with AGPN and acute graft rejection

Demographic, clinical, and biochemical data of patients presenting with AGPN or acute graft rejection are shown in Table 1. The mean age was 49.1 years, and the proportion of male was 48.9% (n = 44). The most common kidney transplant type was deceased kidney (70.0%), followed by living kidney (30.0%), and pancreas-kidney co-transplant (5.6%). Asymptomatic patients with only kidney allograft dysfunction were more common in acute graft rejection group (19.2% vs. 42.1%, P = 0.033). However, DNI values [2.9% (0.0–21.0) vs. 1.9% (0.0–2.9), P < 0.001], WBC counts [(13.1 ± 5.1 x 10^3^/mm^3^) vs. (10.9 ± 2.1 x 10^3^/mm^3^), P = 0.007], neutrophil counts [(9.3 ± 3.6 x 10^3^/mm^3^) vs. (7.2 ± 3.5 x 10^3^/mm^3^), P = 0.008], lymphocyte counts [(2.6 ± 1.5 x 10^3^/mm^3^) vs. (2.0 ± 1.2 x 10^3^/mm^3^), P = 0.050], CRP concentration [60.0 mg/L (9.8–198.0) vs. 30.0 mg/L (7.8–80.0), P = 0.009), and procalcitonin concentration [6.5 mg/dL (0.3–64.0) vs. 0.5 mg/dL (0.0–9.0), P < 0.001] were significantly higher in AGPN group than in acute graft rejection group. However, no differences were observed in the duration of the episodes from the time of kidney transplant, immunosuppressive regimen, hemoglobin concentration, platelet counts, blood urea nitrogen concentration, and creatinine concentration between the two groups.

**Table 1:** Clinical and biochemical characteristics in 90 episodes of acute graft pyelonephritis (AGPN) and acute graft rejection.

Variables	Total (90 episodes)	AGPN (52 episodes)	Acute graft rejection (38 episodes)	P-value
Demographic data				
Age (year)	49.1 ± 8.6	48.6 ± 8.1	49.7 ± 9.4	0.581
Male (%)	44 (48.9)	21 (40.4)	23 (60.5)	0.087
Clinical data				
Transplantation type				
Living	27 (30.0)	13 (25.0)	14 (36.8)	0.251
Deceased	63 (70.0)	39 (75.0)	24 (63.2)	0.251
Pancreas-kidney	5 (5.6)	4 (7.7)	1 (2.6)	0.392
Duration from KT to episode (m)	4.5 (0.06–59.6)	4.6 (0.1–59.6)	4.7 (0.7–48.4)	0.228
Body temperature	38.3 ± 0.5	38.4 ± 0.6	38.2 ± 0.3	0.011
Symptoms				
Graft pain	27 (30.0)	19 (36.5)	8 (21.1)	0.162
Chills	22 (24.2)	9 (17.3)	13 (34.2)	0.084
Cystitis	15 (16.7)	14 (26.9)	1 (2.6)	0.003
Asymtomatic	26 (28.9)	10 (19.2)	16 (42.1)	0.033
Immunosuppressive regimen				
Tacrolimus	52 (57.8)	31 (59.6)	21 (55.3)	0.829
Cyclosporine	37 (41.1)	20 (38.5)	17 (44.7)	0.665
Mycophenolate mofetil	51 (56.7)	32 (61.5)	19 (50.0)	0.291
Azathioprine	39 (43.3)	20 (38.5)	19 (50.0)	0.291
Laboratory data				
Hb (g/dL)	11.1 ± 1.3	11.1 ± 1.6	11.2 ± 1.0	0.819
WBC (10^3^/mm^3^)	12.2 ± 4.2	13.1 ± 5.1	10.9 ± 2.1	0.007
Neutrophil (10^3^/mm^3^)	8.4 ± 3.7	9.3 ± 3.6	7.2 ± 3.5	0.008
Lymphocyte (10^3^/mm^3^)	2.3 ± 1.4	2.6 ± 1.5	2.0 ± 1.2	0.050
DNI (%)	2.5 (0.0–21.0)	2.9 (0.0–21.0)	1.9 (0.0–2.9)	<0.001
Platelet (10^3^/ mm^3^)	247.1 ± 75.5	241.1 ± 73.0	255.7 ± 79.1	0.386
CRP (mg/L)	40.0 (7.8–198.0)	60.0 (9.8–198.0)	30.0 (7.8–80.0)	0.009
PCT (mg/dL)	2.5 (0.0–64.0)	6.5 (0.3–64.0)	0.5 (0.0–9.0)	<0.001
BUN (mg/dL)	24.1 ± 13.2	23.4 ± 16.5	24.9 ± 6.3	0.551
Cr (mg/dL)	1.6 ± 1.0	1.4 ± 1.2	1.8 ± 0.7	0.148

Values are expresses as mean ± SD or median (range) or number (percentage). BUN, blood urea nitrogen; Cr, creatinine; CRP, C-reactive protein; DNI, delta neutrophil index; Hb, hemoglobin; KT, kidney transplant; PCT, procalcitonin; WBC, white blood cell; m, months

### Microbiological results

Microbiological isolates were confirmed in the all 52 episodes of AGPN. The most frequently isolated pathogen were Escherichia coli (n = 26), Pseudomonas aeruginosa (n = 11), and Klebsiella pneumonia (n = 4) (Table 2). In contrast, urine cultures were performed in 31 episodes of 38 episodes with acute graft rejection, and any microbiological isolates were not confirmed in them.

**Table 2:** Organism causing acute graft pyelonephritis in kidney transplant recipients.

Causative microorganisms	Number of positive cultures	Frequency (%)
Gram-negative rods		
Escherichia coli	26	50.0
Pseudomonas aeruginosa	11	21.2
Enterobacter cloacae	3	5.8
Klebsiella pneumoniae	4	7.7
Klebsiella oxytoca	3	5.8
Serratia marcescens	1	1.9
Stenotrophomonas maltophilia	1	1.9
Gram-positive cocci		
Enterococcus faecium	2	3.8
Staphylococcus aureus	1	1.9
Total	52	100

### Associations between DNI values and other parameters in patients with AGPN

There were significant positive correlations between DNI values and procalcitonin concentration (r = 0.45, P = 0.001) while there were no correlation DNI values and WBC counts, neutrophil counts, platelet counts and CRP concentration.

### Comparison of DNI values according to the degree of bacterial infection

**Fig 1:**
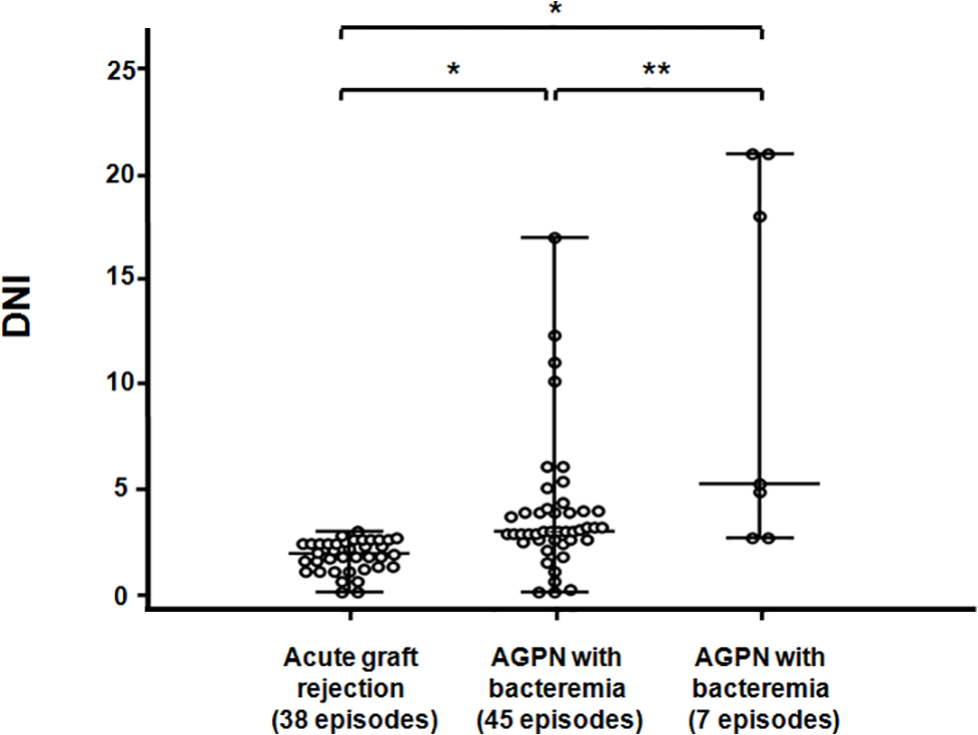
Scattered plots of delta neutrophil index (DNI) values in the three groups: acute graft rejection, AGPN without sepsis, and AGPN with sepsis. Bar and error bar show the median and range, respectively. *P < 0.001 vs. acute rejection group, **P = 0.031 vs. AGPN group without bacteremia. AGPN, acute graft pyelonephritis.

To evaluate the influence of the degree of bacterial infection on DNI values, we divided AGPN group into two subgroups; with bacteremia (7 episodes), without bacteremia (45 episodes). Compared to the AGPN group without bacteremia, DNI values were significantly increased in the AGPN group with bacteremia [5.2% (2.6–21.0) vs. 2.9% (0.0–17.0), P = 0.031]. Additionally, DNI values were also significantly higher in AGPN group without bacteremia than in acute graft rejection group [2.9% (0.0–17.0) vs. 1.9% (0.0–2.9), P < 0.001, respectively] (Fig 1). On note, the AGPN group had significantly higher DNI values than low urinary tract infection group (S1 Fig).

### Comparison of parameters to predict AGPN

**Fig 2:**
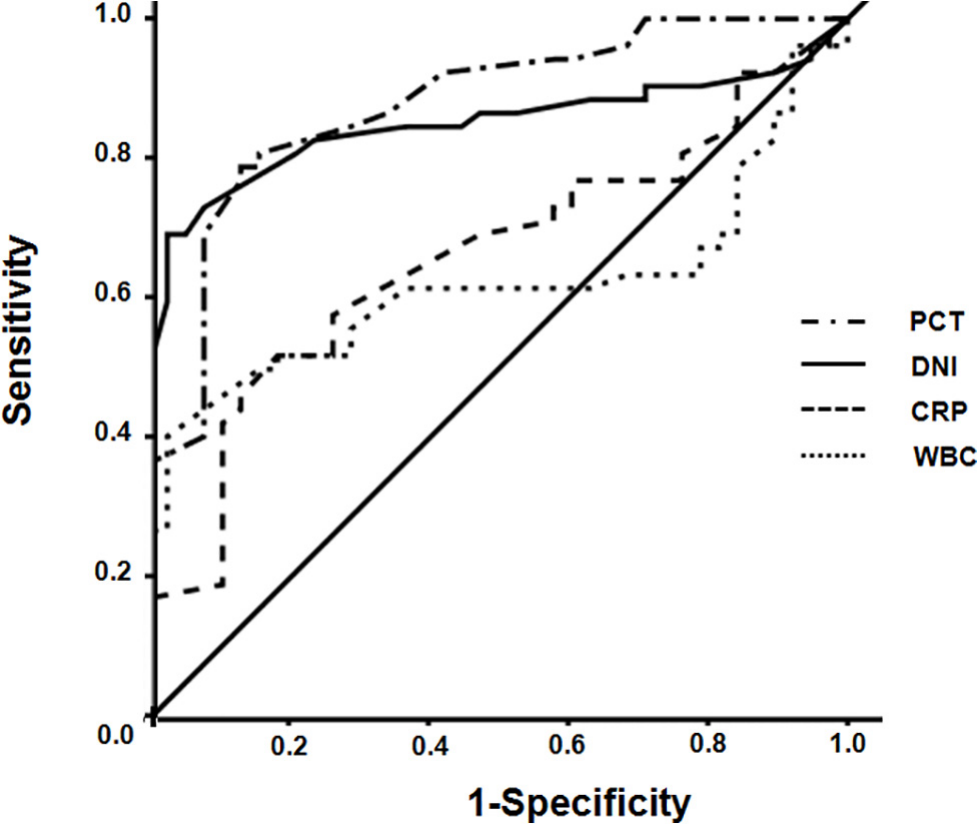
The receiver operating characteristic curves for white blood cells, delta neutrophil index, C-reactive protein, and procalcitonin with respect to the prediction of AGPN. AGPN, acute graft pyelonephritis.

To compare of the ability of the WBC count, DNI value, CRP concentration, and procalcitonin concentration to predict AGPN, we used ROC curve analysis with AUC. The AUC of procalcitonin concentration and DNI values were comparable [(0.88; 95% confidence interval [CI], 0.79–0.94) vs. (0.85; 95% CI, 0.76–0.92), P = 0.311]. Additionally, the AUC of DNI values was also larger than that of WBC counts [(0.85; 95% CI, 0.76–0.92) vs. (0.64; 95% CI, 0.53–0.75), P = 0.002] and CRP concentration [(0.85; 95% CI, 0.76–0.92) vs. (0.66; 95% CI, 0.55–0.76), P = 0.027], suggesting that DNI value was a better predictor of AGPN than WBC count and CRP concentration (Table 3 and Fig 2). Even after excluding episodes of AGPN with bacteremia, the AUC of DNI values was also larger than that of WBC counts and CRP concentration (S1 Table).

**Table 3:** Receiver operating characteristic (ROC) curve values of predictive factors for acute graft pyelonephritis.

Variables	AUC (95%CI)	Sensitivity (%) (95% CI)	Specificity (%) (95% CI)	Cutoff point
WBC (10^3^/mm^3^)	0.64 (0.53–0.75)	61.54 (47.0–74.7)	75.86 (56.5–89.7)	11.3
DNI (%)	0.85 (0.76–0.92)	69.23 (54.9–81.3)	97.37 (86.1–99.6)	2.7
CRP(mg/L)	0.66 (0.55–0.76)	51.92 (37.6–66.0)	81.58 (65.7–92.2)	56.0
PCT (mg/dL)	0.88 (0.79–0.94)	78.85 (65.3–88.9)	86.84 (71.9–95.5)	1.0

AUC, area under the ROC curves; CRP, C-reactive protein; DNI, delta neutrophil index; PCT, procalcitonin; WBC, white blood cell

### Optimal DNI cutoff value to predict AGPN

To obtain the optimal DNI cutoff value to predict AGPN, we also used ROC curve analysis with AUC. The best cutoff value for DNI to predict AGPN was 2.7% with a sensitivity and specificity of 69.23% (95% CI, 54.9–81.3) and 97.37% (CI, 86.1–99.6), respectively (Table 3). In patients with DNI value < 2.7%, 36 episodes (69.2%) were acute graft rejections and 16 episodes (30.8%) were AGPN, while in patients with DNI value ≥ 2.7%, 2 episodes (5.3%) were acute graft rejections and 36 episodes (94.7%) were AGPN (Table 3). Among patients with AGPN or acute graft rejection, those with DNI ≥ 2.7% were found to have a higher risk of AGPN than those with DNI < 2.7% (odds ratio [OR], 40.50; 95% CI, 8.68–189.08, P < 0.001) (Table 4).

**Table 4:** Prevalence of acute graft pyelonephritis (AGPN) and acute rejection according to a DNI cutoff of 2.7%.

	DNI < 2.7% (n = 52)	DNI ≥ 2.7% (n = 38)	Odds ratio for infection
AGPN (%)	16 (30.8)	36 (94.7)	40.50 (8.68–189.08)
Acute rejection (%)	36 (69.2)	2 (5.3)	40.50 (8.68–189.08)

DNI, delta neutrophil index

### Independent predictive value of DNI for AGPN

Among patients with AGPN or acute graft rejection episodes, univariate logistic regression analysis revealed an increase of AGPN episodes in those with increased DNI value (odd ratio [OR], 2.76; 95% CI, 1.60–4.77, *P* < 0.001). Additionally, due to skewed distribution, WBC counts, neutrophil counts and lymphocyte counts were log-transformed in regression analyses. Log-transformed neutrophil counts (OR, 4.65, 95% CI, 1.49–14.56, P = 0.008), DNI values (OR, 2.86; 95% 1.65–4.95, *P* < 0.001), CRP concentration (OR, 1.02; 95% CI, 1.01–1.04, P = 0.006) and procalcitonin concentration (OR, 1.47; 95% CI, 1.19–1.82, P < 0.001) were found to be associated with a higher risk of AGPN. Lastly, the impact of DNI value on AGPN remained significant even after adjustment for age, sex, log WBC count, log neutrophil count, log lymphocyte count, DNI value, CRP concentration, and procalcitonin concentration (OR, 4.32; 95% CI, 1.81–10.34, P < 0.001) (Table 5). Additionally, even though episodes with bacteremia were excluded, DNI was also a significant independent factor for predicting AGPN (S2 Table).

**Table 5:** Univariate and multivariate analysis of independent predictor variables for acute graft pyelonephritis.

Variables	Univariate		Multivariate	
	OR (95% CI)	*P*-value	OR (95% CI)	P-value
Age (per 1 year increase)	0.99 (0.94–1.04)	0.576	0.93 (0.86–1.02)	0.111
Male (versus female)	0.44 (0.19–1.04)	0.061	0.70 (0.15–3.15)	0.637
Log WBC (/mm^3^)	2.77 (0.80–9.58)	0.108	0.17 (0.01–6.80)	0.348
Log Neutrophil (10^3^/mm^3^)	4.65 (1.49–14.56)	0.008	3.66 (0.21–63.00)	0.372
Log Lymphocyte (10^3^/mm^3^)	1.87 (0.89–3.90)	0.097	0.67 (0.15–3.06)	0.600
DNI (per 1% increase)	2.86 (1.65–4.95)	<0.001	4.32 (1.81–10.34)	0.001
CRP (mg/L)	1.02 (1.01–1.04)	0.006	1.01 (0.98–1.05)	0.369
PCT (mg/dL)	1.47 (1.19–1.82)	<0.001	1.58 (1.16–2.1)	0.003

OR, odd ratio; CRP, C-reactive protein; DNI, delta neutrophil index; PCT, procalcitonin; WBC, white blood cell

### The impact of DNI on kidney allograft function in AGPN

To examine the impact of DNI on kidney allograft function in AGPN, the episodes with kidney allograft dysfunction and their outcomes were divided according to tertile of DNI values as follow: tertile 1, DNI < 2.8%; tertile 2, 2.8%–3.8%; tertile 3, > 3.8%. Among 52 episodes with AGPN, 21 (40.4%) episodes had kidney allograft dysfunction. The proportion of episodes with kidney allograft dysfunction was significantly higher in the highest tertile of DNI (P for trend = 0.004). Additionally, as DNI values increased, kidney allograft outcome was significantly poor at one month after antibiotics therapy among them (P for trend = 0.016) (Table 6).

**Table 6:** The impact of DNI on kidney allograft function in acute graft pyelonephritis.

	DNI (%)			
	< 2.8 (n = 17)	2.8–3.8 (n = 17)	> 3.8 (n = 18)	P-value for trend
Episodes without kidney allograft dysfunction^a^ (%)	15 (88.2)	10 (58.8)	6 (33.3)	0.004
Episodes with kidney allograft dysfunction^a^ (%)	2 (11.8)	7 (41.2)	12 (66.7)	
Kidney allograft outcome ^b^				0.016
Good (%)^c^	2 (100)	1 (14.3)	1 (8.3)	
Moderate (%)^d^	0 (0.0)	4 (57.1)	4 (33.3)	
Poor (%)^e^	0 (0.0)	2 (28.6)	7 (58.3)	

^a^Increase in the serum creatinine concentration of 25% over baseline levels

^b^The kidney allograft outcomes were evaluated at 1 month after starting antibiotic therapy among episodes with kidney allograft dysfunction

^c^> 25% reduction in serum creatinine

^d^ < 25% reduction in serum creatinine

^e^No reduction in serum creatinine

## Discussion

This study showed that DNI, which reflects the number of circulating granulocyte precursors in the blood, was an effective marker to differentiate between AGPN and acute graft rejection in renal transplant recipients. Our finding suggests that kidney transplant recipients with elevated DNI value are found to be at a higher risk for infection than those with decreased DNI value.

The value of quantitative urine culture in the diagnosis of pyelonephritis has been demonstrated in early studies in which this method was able to discriminate between true urinary tract infections and contaminated urine specimens. In other words, bacterial counts of 10^5^ CFU per mL or higher in midstream urine cultures were predictive of bladder bacteriuria in women who are asymptomatic or have pyelonephritis, whereas lower counts were more likely to be associated with contamination [27, 28]. However, later studies showed that women with symptoms of cystitis often had lower colony counts [27, 29]. Therefore, we included < 10^5^ CFU per mL as the threshold for the diagnosis of AGPN.

In kidney transplant recipients, urinary tract infection (UTI) is the most common form of bacterial infection [2, 3]. As in the non-transplant population, painful voiding, urgency, frequency, and occasional pain of the lower abdomen and hematuria are the leading symptoms of UTI after kidney transplant, at times accompanied by fever. However, as the transplanted organ has been denervated during transplantation, and as the recipient most often is under immunosuppression, the UTI symptoms can be masked. Although kidney transplant recipients have virtually no symptoms of UTI, as the kidney allograft is usually placed in the right or left iliac fossa, AGPN must be considered if lower right or left abdominal quadrant pain plus fever develops in the recipients [19]. However, acute graft rejection can also produce fever and pain on the transplanted kidney area, but, unnecessary antibiotic treatment could be toxic for graft itself in these patients [20]. Conversely, in patients with infection, immunosuppressant therapy to prevent acute graft rejection might exacerbate infection [21]. Therefore, the distinction between AGPN and acute graft rejection is very important for the prompt management of these patients in clinical practice. Although various markers such as WBC count and CRP concentration are commonly used to identify bacterial infection, their validity is limited in kidney transplant recipients undergoing immunosuppressive therapy [8, 22]. For example, Immunosuppressant agent such as corticosteroids can raise the WBC or neutrophil count [23] and MMF is associated with lower CRP concentration in kidney, cardiac transplant recipients and patients with IgA nephropathy [24–26]. Furthermore, CRP concentration or WBC count have been shown to increase in pediatric transplantation patients with acute rejection or bacterial infection [22]. Although procalcitonin concentration seem to be unaffected by corticosteroid therapy, unlike many other markers of immune system activity, it is less available than CRP concentration and its cost-effectiveness is uncertain [7, 20].

In previous studies, the proportion of IGs was better correlated with positive blood culture results and infection than the WBC count [13], whereas the level of IGs was suggested as a predictor of neonatal sepsis [30]. However, as it is difficult to measure IGs accurately, their diagnostic value remains controversial. To overcome these limitations, DNI value, calculates the difference between leukocyte differentials measured in the MPO channel and those measured in the nuclear lobularity channel, was designed and found to be a reliable and reproducible method reflecting blood IGs [15]. As DNI value is automatically indicated as part of CBC test with no additional charge to the patients, it is considered an attractive maker for use in the clinical practice where cost effectiveness is crucial. Recently, Pyo et al. investigated the role of DNI value in the discriminating between disease flare-up and infection in patients with systemic lupus erythematous patients [17]. Although leucopenia and leukocytosis were observed in some patients, the latter because of glucocorticoid usage, DNI value reflected the proportion of IGs regardless of WBC count, and as the WBC count can be affected by other non-infectious conditions, DNI value was also more valuable than the WBC count in cases of infection.

In line with recent finding, the results from the present study clearly showed that DNI values were higher in patients with AGPN compared to those with acute graft rejection. In addition, we observed that DNI values significantly correlated with procalcitonin concentration in AGPN group. Moreover, ROC analysis showed that the diagnostic value of DNI for AGPN was comparable to procalcitonin concentration and it was superior to WBC count and CRP concentration. Furthermore, we proposed a novel cutoff value of DNI at 2.7% to predict infection, with a sensitivity of 69.2% and a specificity of 97.4% which is comparable to the 2.8% value reported by Pyo et al [17]. Lastly, increased DNI values were independently predictive of infection in multivariate analyses even after adjustment for other markers used to confirm bacterial infection. Taken together, although DNI was low sensitivity to discriminate between AGPN and acute graft rejection in this study, it may be more helpful to use DNI in combination with other maker, such as procalcitonin. Higher DNI was correlated with poor allograft outcome at one month after antibiotic treatment. Attractively, DNI had the added benefit of being simple and economic.

Our study had several limitations. First, it was an uncontrolled retrospective study based on a small population who underwent kidney transplant at a single center. Second, protocol renal allograft biopsy was not performed. Thus, the episodes of AGPN with asymptomatic or atypical clinical presentation might not be included in this study. For this reason, our results should be regarded as preliminary, and further prospective studies including protocol renal allograft biopsy are needed. Third, although we excluded episodes occurring in recipients who had neutropenia resulting from drugs inducing bone marrow suppression, DNI value can be influenced by immunosuppressive drugs. Because they may reduce the hematopoietic function of bone marrow, resulting in relatively decreased changes in the number of IGs. In this regard, the sensitivity of DNI to predict AGPN was not high in this study. Lastly, as DNI was measured at the time that patients presented with AGPN or acute graft rejection, DNI alterations along a longer course of each disease are still unclear.

In conclusion, the present study demonstrated that a DNI value above 2.7% was an independent predictive marker for AGPN in patients in whom a differential diagnosis between AGPN and acute graft rejection is needed. Therefore, these finding suggest that DNI may be a useful marker in the management of these patients.

## Supporting Information

S1 Fig Scattered plots of delta neutrophil index (DNI) values between AGPN group and low urinary tract infection group. Bar and error bar show the median and range, respectively. There were 59 kidney transplant recipients with 62 episodes of low urinary tract infection during study follow-up period. *P < 0.001 vs. low urinary tract infection group.
(TIF)

S1 Table Receiver operating characteristic (ROC) curve values of predictive factors for acute graft pyelonephritis without bacteremia. (DOC)

S2 Table Univariate and multivariate analysis of independent predictor variables for acute graft pyelonephritis without bacteremia. (DOC)
