# Supplementary figures and images for: Delta Neutrophil Index as a Marker for Differential Diagnosis between Acute Graft Pyelonephritis and Acute Graft Rejection
Source: PLoS One. 2015 Aug 14;10(8):e0135819. doi: 10.1371/journal.pone.0135819 (PMC4537133; doi:10.1371/journal.pone.0135819)

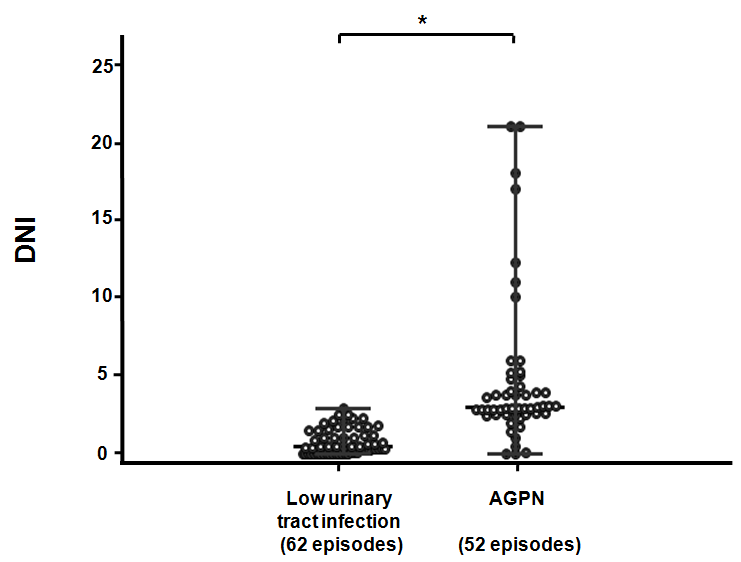

Supplement: S1 Fig — Bar and error bar show the median and range, respectively. There were 59 kidney transplant recipients with 62 episodes of low urinary tract infection during study follow-up period. *P < 0.001 vs. low urinary tract infection group. (TIF) [file pone.0135819.s001.tif]
