# Supplementary material for: Delta Neutrophil Index as a Marker for Differential Diagnosis between Acute Graft Pyelonephritis and Acute Graft Rejection
Source: PLoS One. 2015 Aug 14;10(8):e0135819. doi: 10.1371/journal.pone.0135819 (PMC4537133; doi:10.1371/journal.pone.0135819)
Supplement: S1 Table — (DOC) [file pone.0135819.s002.doc]

S1 Table. Receiver operating characteristic (ROC) curve values of predictive factors for acute graft pyelonephritis without bacteremia

| Variables | AUC  (95%CI) | Sensitivity (%)  (95% CI) | Specificity (%)  (95% CI) | Cutoff  point |
| --- | --- | --- | --- | --- |
| WBC (10^3^/mm^3^) | 0.60 (0.48 - 0.71) | 46.67 (31.7 - 62.1) | 86.21 (68.3 - 96.1) | 13.0 |
| DNI (%) | 0.83 (0.73 - 0.90) | 68.89 (53.4 - 81.8) | 97.37 (86.2 - 99.9) | 2.7 |
| CRP(mg/L) | 0.64 (0.53 - 0.74) | 44.44 (29.6 - 60.0) | 86.84 (71.9 - 95.6) | 60 |
| PCT (mg/dL) | 0.88 (0.79 - 0.94) | 77.78 (62.9 - 88.8) | 86.84 (71.9 - 95.6) | 1.0 |

AUC, area under the ROC curves; CRP, C-reactive protein; DNI, delta neutrophil index; PCT, procalcitonin; WBC, white blood cell
