# Supplementary material for: Delta Neutrophil Index as a Marker for Differential Diagnosis between Acute Graft Pyelonephritis and Acute Graft Rejection
Source: PLoS One. 2015 Aug 14;10(8):e0135819. doi: 10.1371/journal.pone.0135819 (PMC4537133; doi:10.1371/journal.pone.0135819)
Supplement: S2 Table — (DOC) [file pone.0135819.s003.doc]

S2 Table. Univariate and multivariate analysis of independent predictor variables for acute graft pyelonephritis without bacteremia

| Variables |  | Univariate | |  | Multivariate | |
| --- | --- | --- | --- | --- | --- | --- |
|  |  | OR (95% CI) | P-value |  | OR (95% CI) | P-value |
| Age (per 1 year increase) |  | 0.99 (0.94 - 1.04) | 0.604 |  | 0.94 (0.86 - 1.02) | 0.124 |
| Male (versus female) |  | 0.52 (0.22 - 1.25) | 0.146 |  | 0.71 (0.16 - 3.18) | 0.650 |
| Log WBC (/mm^3^) |  | 2.11 (0.60 - 7.40) | 0.243 |  | 0.17 (0.01 - 6.69) | 0.347 |
| Log Neutrophil (10^3^/mm^3^) |  | 4.60 (1.11 - 10.96) | 0.032 |  | 3.41 (0.19 - 60.09) | 0.401 |
| Log Lymphocyte (10^3^/mm^3^) |  | 1.81 (0.86 - 3.81) | 0.118 |  | 0.70 (0.15 - 3.30) | 0.652 |
| DNI (per 1% increase) |  | 2.74 (1.58 - 4.75) | <0.001 |  | 4.22 (1.75 - 10.14) | 0.001 |
| CRP (mg/L) |  | 1.02 (1.01 - 1.04) | 0.013 |  | 1.01 (0.98 - 1.05) | 0.395 |
| PCT (mg/dL) |  | 1.45 (1.17 - 1.80) | 0.001 |  | 1.56 (1.15 - 2.12) | 0.004 |

OR, odd ratio; CRP, C-reactive protein; DNI, delta neutrophil index; PCT, procalcitonin; WBC, white blood cell
